# The Multifaceted Role of P2X7R in Microglia and Astrocytes

**DOI:** 10.1007/s11064-025-04502-y

**Published:** 2025-07-21

**Authors:** Martina Bedetta, Paola Pizzo, Annamaria Lia

**Affiliations:** 1 Department of Biomedical Sciences, University of Padova, Padua, Italy; 2 Neuroscience Institute, Italian National Research Council (CNR), Padua, Italy; 3 Study Centre for Neurodegeneration (CESNE), University of Padova, Padua, Italy

**Keywords:** P2X7R, Glia, Inflammation, eATP

## Abstract

The purinergic P2X7 receptor (P2X7R) is a unique ATP-gated ion channel that requires unusually high concentrations of extracellular ATP (eATP) for activation, making it a sensor of cellular stress and injury in the central nervous system. This review provides a comprehensive overview of P2X7R expression and function in glial cells, with a particular focus on microglia and astrocytes. We first outline the molecular characteristics of P2X7R and its distribution in brain cell types. In microglia, P2X7R regulates a broad spectrum of processes, including phagocytosis, autophagy, proliferation, and ultimately cell death, underscoring its dual role in neuroprotection and neurotoxicity. In astrocytes, P2X7R contributes to gliotransmission and inflammatory signaling, influencing neuronal excitability and synaptic function. We further explore the role of P2X7R in the context of both Alzheimer’s disease and epilepsy, where a dysregulated eATP-P2X7R signaling axis exacerbates neuroinflammation and glial dysfunction. Understanding the cell-specific roles of P2X7R in physiology and pathology provides new insights into glial biology and highlights its potential as a therapeutic target in brain diseases.

## Introduction

### The Purinergic Receptor P2X7

Brain function requires an intercellular communication system capable of integrating vascular, glial, immune, and neural components into a dynamic yet tightly controlled network, with all these elements being part of the so-called brain active *milieu* [1, 2]. Extracellular ATP (eATP) is a molecular signal that uniquely serves for this task. ATP and its metabolite adenosine are produced and released by cells in all tissues and are crucial neuromodulators in the central nervous system (CNS) [3, 4]. The signaling pathways triggered by ATP (and adenosine) involve the activation of purinergic receptors and support a broad spectrum of functions, spanning from rapid synaptic transmission to long-term processes such as development. Different mechanisms account for ATP release including: transmembrane diffusion via ATP-permeable channels, active transport and exocytosis [5–7]. The concept of purinergic nerves was first described in 1972 by Geoffrey Burnstock, who later described purinergic receptors in 1976 [8, 9], and represent an ancient form of intercellular communication, with ATP sensitivity and purinoceptors emerging early in evolution, being present already in single-cell protozoa and algae, reflecting its fundamental and highly conserved role in cellular signaling [10]. They are divided into two main subclasses: P1 and P2 receptors. While P1 receptors are activated by adenosine and coupled to G proteins, P2 receptors are divided into ionotropic P2X receptors and metabotropic P2Y receptors [11]. P2X receptors are ATP-gated cationic channels located in the plasma membrane (PM) and represent the earliest receptor to appear in evolution. They are composed of three subunits, each of which has two transmembrane domains [12–14]. P2X7 receptor (P2X7R) is one of the most investigated purinergic receptors. It usually assembles as a homotrimer [15] and, after ATP binding, it allows the influx of Na^+^ and Ca^2+^ and the efflux of K^+^ [16]. The resulting increase in intracellular Ca²⁺ concentration activates various downstream cellular processes. One key pathway involves calcineurin, a Ca²⁺-dependent phosphatase that activates NFAT, a transcription factor that upon nucleus translocation upregulates inflammatory gene expression [17]. In parallel, P2X7R stimulation can activate MAPK pathways (ERK1/2, p38, JNK), CaMKII/CREB signaling, and calpain proteases, all of which influence cell cycle progression and survival [18], the release of TNF-α [19] and cytoskeletal remodeling [20]. Of note, differently from other P2 receptors, P2X7R has a low affinity to ATP, requiring high levels of eATP (> 0.1 mM) for its activation [21]. eATP reaches high concentrations (in the order of hundred micromolar) in inflamed conditions, while in healthy tissue its concentration is kept in the low nanomolar range [22, 23]. Accordingly, P2X7R results activated only when high eATP concentrations are reached, such as in diseased tissue, and here it has a crucial role in sustaining inflammation by inducing the assembly of the NLRP3 inflammasome and the subsequent IL-1β production. Several mechanisms have been proposed to contribute to the P2X7R-mediated inflammasome activation. Different works point to a crucial role of intracellular K^+^ depletion, through P2X7R opening, as a driver of inflammasome activation [24–26]. This K^+^ efflux model has been questioned by Pelegrin and co-workers [27] that instead support pannexin-1 (Panx1) as a key molecule upstream of P2X7R activation. Panx1 physically interacts with P2X7R and P2X7R opening leads to Panx1 activation. In this status, Panx1 acts as a conduit for intracellular ATP release, therefore increasing eATP concentration and further sustaining P2X7R activation. Interestingly, by blocking Panx1 in macrophages, the release of IL-1β was blocked in these cells, without altering cellular K^+^ depletion. As a functional (but debated) model, the authors propose that P2X7R activation leads to Panx1 opening and ATP entry, a signal that may directly lead to caspase-1 activation and consequent IL-1β maturation/release [28]. Notably, under inflammatory conditions, characterized by chronically elevated eATP levels, P2X7R may become permeable to larger molecules - up to 900 Da - including ATP itself [29], thereby amplifying the inflammatory response. Despite these controversies, activation of P2X7R is required for IL-1β production and release, indeed several selective P2X7R antagonists are able to reduce the release of this cytokine in inflammatory conditions [30].

### P2X7R Distribution in Brain Cells

The expression of P2X7R in the different cells of the CNS is still a matter of debate. In 1996 two independent groups firstly demonstrated the functional expression of P2X7R (originally called P2Z receptor) in microglial cells [31, 32]. Successively, another study pointed to the presence of P2X7R-mRNA exclusively in microglia where it is upregulated after stroke lesion, namely middle cerebral artery occlusion (MCAO), in rodents [33]. Since then, this conclusion has been questioned and many studies using advanced pharmacological tools and new animal models supported the presence of neuronal P2X7R in different brain regions [34–36]. Indeed, a functional role of P2X7R in modulating neuronal differentiation and neuronal physiology has been reported, while in pathological conditions neuronal P2X7R seems to contribute in spreading neuronal cell death [37]. On the other hand, although not excluding the presence of P2X7R in neurons, Illes and co-workers supported the view of astrocytic and microglial P2X7Rs as primary targets of high eATP concentration in the brain [38, 39]. The presence of P2X7R in glial cells of both CNS and peripheral nervous systems has been confirmed by using both pharmacological and receptor expression studies. A very elegant study was performed in 2018 by Kaczmarek-Hajek and co-workers by using a newly developed genetically modified mice expressing a P2X7R fused with a green-fluorescent tag. The authors confirmed the presence of P2X7R predominantly in microglia and oligodendrocytes and in a subpopulation of astrocytes, while they failed to assess neuronal P2X7R expression in the hippocampus [40].

In this review we will summarize the most recent discoveries on the role of P2X7R in microglia and astrocytes with a particular focus on the role of P2X7R in the context of Alzheimer’s disease (AD) and epilepsy.

### Microglial P2X7R and Inflammatory Response

Microglia, which constitute approximately 5–20% of the total glial cell population [41], are the resident immune cells of the brain and play a key role in maintaining CNS homeostasis and injury response, by clearing debris and detecting extracellular signals by phagocytosis and inflammatory signaling pathways, respectively [42]. Microglia activation reflects a dynamic continuum between a pro-inflammatory and an anti-inflammatory and reparative state. Microglia is activated by stimuli such as IFN-γ and LPS, release cytokines like TNF-α, IL-1β, IL-6, IL-12, and chemokines that promote inflammation, oxidative stress, and neuronal damage. Conversely, if stimulated by IL-4 and IL-13, microglia produce anti-inflammatory and neurotrophic factors that support tissue repair and neuronal survival [43]. P2X7R plays a pivotal role in regulating microglia polarization in response to different kinetics of eATP rise. Indeed, transient eATP increases activate P2X7R, promoting both pro-inflammatory signaling and anti-inflammatory markers like Arg-1, suggesting a mixed phenotype [44]. However, chronic P2X7R activation by high, sustained eATP or 3'-O-(4-benzoyl)benzoyl adenosine 5'-triphosphate (BzATP, a potent P2X7R agonist) levels shifts microglia toward a pro-inflammatory state, suppressing phagocytosis and exacerbating neuronal damage [45]. Conversely, agents that reduce eATP concentrations, like astragalus polysaccharides, have been shown to promote microglia anti-inflammatory effects [46]. Moreover, sustained activation of microglial P2X7R has been linked to increased production of reactive oxygen species (ROS), including hydrogen peroxide, contributing to oxidative stress and neurotoxicity [47].

### Microglial P2X7R and Microparticle Release

P2X7R is also a key regulator of non-apoptotic membrane blebbing and microparticles (MPs) release [48, 49]. MPs, including exosomes, microvesicles (MVs), and other extracellular vesicles, represent essential vehicles for cellular communication, by carrying signaling molecules like cytokines, neurotransmitters, and growth factors to target different cells [50]. In murine microglia [51, 52], but not in cultured human microglia [53], the activation of P2X7R causes cytoskeletal rearrangements that lead to dynamic membrane blebbing, a controlled and reversible process that causes PM protrusions. For instance, this process facilitates the release of IL-1β in primary mouse microglia [51]. In particular, activation of P2X7R triggers the rapid translocation of phosphatidylserine and acid sphingomyelinase to the outer leaflet of the PM, leading to the formation of MVs (40–80 nm) containing IL-1β [51, 54]. This mechanism offers a key advantage in setting the inflammatory response: it protects IL-1β from extracellular degradation and dilution, while enabling its targeted delivery to recipient cells expressing IL-1β receptors, thereby regulating the inflammatory signaling pathway [54, 55]. An interesting work investigating the role of P2X7R-mediated microglial MV release in neurotransmission, shows that pre-exposure of cultured hippocampal neurons to microglial MVs induces an increase in the frequency of mini excitatory postsynaptic currents (mEPSCs), consistent with an enhanced release probability of neurotransmitters from presynaptic terminals. These findings were further supported by in vivo experiments, which confirmed an increase in mEPSCs after microglial MV injection into rat primary visual cortex [56]. Noteworthy, content-depleted MVs were still able to increase mEPSC frequency, suggesting a role for MV surface components in promoting exocytosis. Indeed, microglia-derived MVs interact with neuronal membranes and modulate sphingomyelin metabolism, whose products are involved in facilitating neurotransmitter release [57–60]. MPs may also carry mitochondria that are extracellularly released [51]. Mitochondrial transfer is involved in both tissue repair [56] and brain homeostasis [61]. In the CNS, mitochondrial transfer has been documented between neurons and astrocytes [61–63], as well as between astrocytes and microglia, both in vitro and in vivo [64, 65]. Di Virgilio’s group insightfully investigated how P2X7R expression/activation impacts on mitochondria release and microglia-to-microglia mitochondrial transfer. By employing primary microglia from wild-type (WT) or P2X7R knockout (KO) mice, as well as N13 microglia cells with high or low P2X7R expression, they demonstrated that P2X7R expression in donor cells strongly influenced both basal and eATP-induced MP release and their uptake by recipient cells. They thus showed that MPs transfer mitochondria, P2X7R itself, and NLRP3 inflammasome components to recipient cells in a P2X7R-dependent manner. Notably, MPs from highly P2X7R-expressing cells restored key P2X7R functions, such as reversible PM permeabilization, in low P2X7R-expressing cells [66].

### Effect of P2X7R on Microglial Phagocytosis and Autophagy

Although many studies focused on the role of P2X7R in the presence of high eATP concentrations, some groups also reported a role for P2X7R in the regulation of microglia phagocytosis in basal conditions. Initial evidence for the role of unstimulated P2X7R in phagocytosis came from studies in human monocytes, where the blocking of the receptor with antibodies, or its silencing through siRNAs, significantly reduced the uptake of non-opsonized particles [67, 68] and *E. coli* bacteria [69], respectively. Accordingly, P2X7R overexpression in HEK293 cells increased phagocytosis of apoptotic cells and bacteria, without any ATP stimulation [68]. This finding was later confirmed in human microglia, where the activation of P2X7R with the potent agonist BzATP suppressed uptake of fluorescent *E. coli* bioparticles, while the cell treatment with the receptor antagonist A438079 reversed this effect [53]. These results lead to the hypothesis that P2X7R acts as a scavenger for apoptotic cells and bacteria. The scavenger action of P2X7R in microglia clearance of extracellular debris implies a specific, and only partially understood, molecular mechanism, involving both its extracellular and intracellular protein domains. A specific extracellular region (residues 306–320) mediates its binding to particles, such as beads, bacteria, and apoptotic cells [68], while intracellular interactions with non-muscle myosin heavy chain IIA (NMMHC IIA), a cytoskeletal motor protein, link the receptor to cytoskeleton [67, 70]. ATP stimulation causes the dissociation of NMMHC IIA from the receptor and reduces phagocytosis, suggesting a dynamic switch between phagocytic and pore-forming states [70, 71]. Indeed, the receptor is thought to adopt at least two distinct conformational states: a pore-forming state, associated with pro-inflammatory signaling, and a closed conformation, likely responsible for its scavenger function [72]. Notably, inhibitory binding sites distinct from ATP-binding regions have been identified in the P2X7R, suggesting that selective antagonists can block the inflammatory pore without impairing the scavenger function of the receptor [73, 74]. The autophagic activity of microglia, driven by P2X7R activation, is also a multifaceted and multistage process (see [71] for an extensive review on this topic). Briefly, transient stimulation with high concentrations of eATP activates P2X7R, which induces microglia autophagy with a transient increase in the lipidated marker LC3-II. During this phase, microglia express both pro-inflammatory and anti-inflammatory markers. However, prolonged activation of P2X7R results in an elevated lysosomal pH, which compromises microglia degradative capacity. Consequently, autophagic cargos accumulate inside the cell and are subsequently released in the extracellular space [75, 76]. This process is paralleled by a microglia phenotypic shift toward a predominantly pro-inflammatory state [77].

### The Role of P2X7R in Microglial Proliferation and Death

**Fig. 1:**
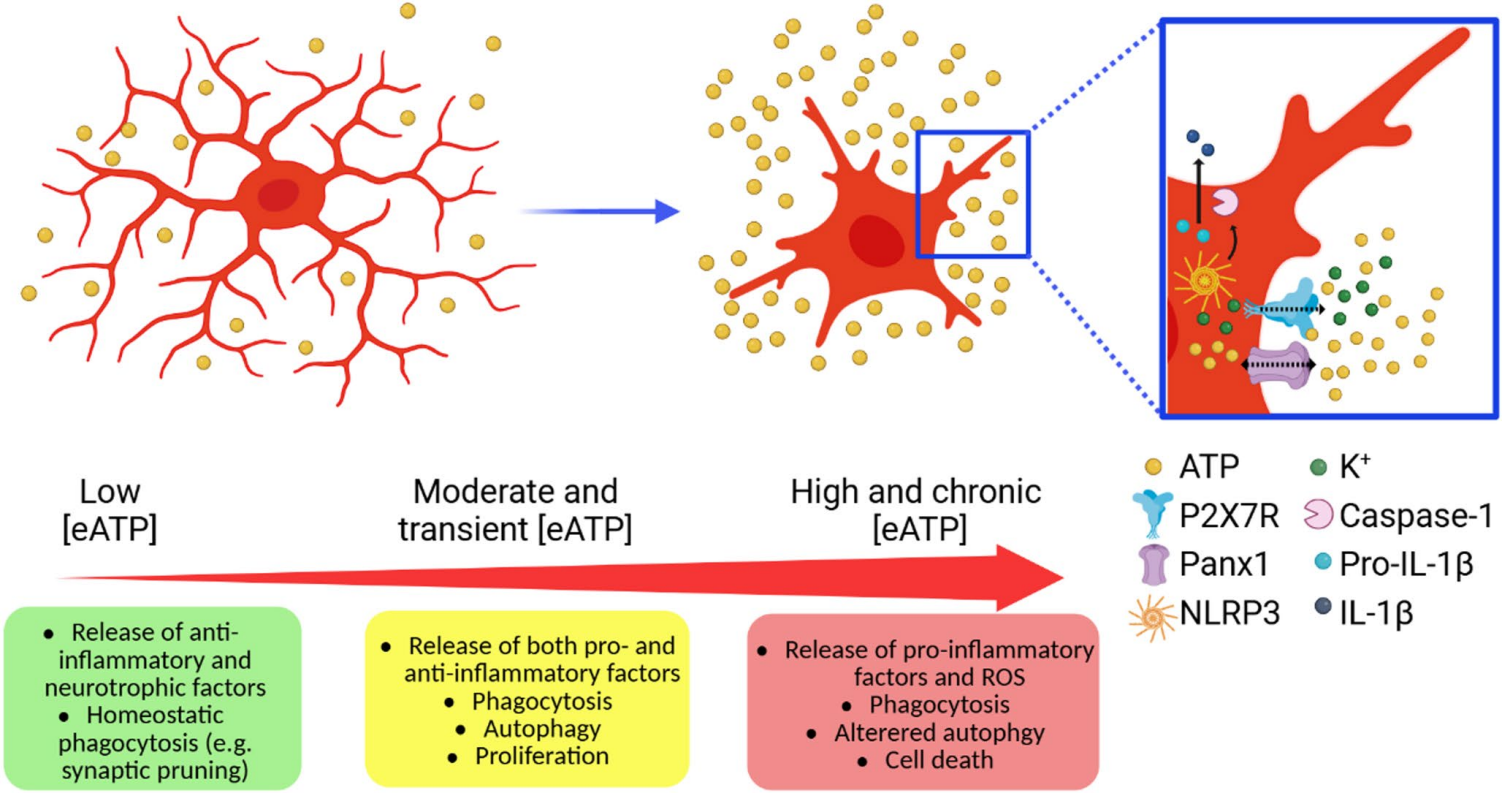
Schematic representation of microglial morphological and functional alterations with respect to increased eATP levels. Created with Biorender.com

P2X7R has a key role in the balance between cell proliferation and death, with its effects depending on cell type, eATP levels and kinetics of receptor activation [78]. In the rat brain, microglia P2X7R expression is detectable as early as embryonic day 16 (E16), with a widespread distribution across the forebrain by postnatal day 30 (P30) [79]. Rigato and co-workers showed that embryonic mouse microglia (E13.5) already express functional P2X7R and, by means of in situ patch-clamp recordings, they demonstrated that microglia P2X7R activation by high concentration of eATP evokes a characteristic biphasic current [80]. Previous in vitro studies suggested that this large biphasic current may result from the interaction between P2X7R and Panx1, and that this interaction drives proliferation and activation of adult microglia [28, 81]. However, microglia obtained from Panx1 KO embryos still exhibit the biphasic current and show no differences in microglial proliferation. Thus, P2X7R seems to regulate the proliferation of embryonic spinal microglia independently from Panx1 [80]. In contrast, sustained activation of P2X7R by high doses of Bz-ATP leads to microglia cell death in the cortex of neonatal mice [82]. These results indicate that P2X7R plays a role already in the early stages of neurodevelopment, potentially helping to prevent excessive microglia proliferation. In the adult brain, microglia density is sustained by a self-renewal process that balances proliferation and apoptosis [83]. Depending on the context, P2X7R has a dual capacity in promoting both proliferation and cell death. Indeed, overexpression of P2X7R alone is sufficient to promote microglia proliferation in primary rat hippocampal cultures. This effect was significantly attenuated by the P2X7R antagonist oxidized ATP (oATP), indicating a direct contribution of the receptor to microglia proliferation [54, 84]. In purified primary cultures of retinal microglia, activation of P2X7R by BzATP significantly increases microglial proliferation and promotes morphological changes typical of microglia activation. This effect was markedly reduced by co-application of the P2X7R antagonist Brilliant Blue G (BBG) [85]. On the other hand, a cytotoxic role for P2X7R in microglia has also been described. Initial studies showed that sustained P2X7R stimulation either by high concentrations of ATP or BzATP [86] or prolonged ATP treatment (e.g., 60 min) [51], leads to cell death in the N9 murine microglial cell line. Accordingly, in primary mouse microglia, high concentration of BzATP (380µM) induces significant cell death. This effect was not observed in P2X7RKO microglia, indicating that P2X7R is essential for BzATP-induced cytotoxicity [82]. Altogether, these data suggest that moderate or transient activation of P2X7R likely promotes microglia proliferation, whereas sustained or intense stimulation of the receptor triggers cell death. In Fig. 1 we summarized the microglial alterations with respect to eATP level variations.

### Astrocytic P2X7R and Gliotransmitter Release

Astrocytes were initially recognized as crucial cells for the maintenance of brain homeostasis but nowadays it is well accepted that they are also actively involved in synaptic transmission, neurovascular coupling and in the regulation of different behavioural states (for extensive reviews on these topics see [87, 88]). Most of the crucial functions exerted by astrocytes rely on gliotransmitter release [89] and it is well known that a pivotal role is exerted by eATP released through both Ca^2+^-dependent exocytosis and non-exocytotic pathways [90–92]. Interestingly, the P2X7R–Panx1 complex provides sites of eATP release in primary spinal cord astrocytes, with a stronger contribution with respect to Connexin-43 (Cx43) hemichannels [93, 94]. Besides its involvement in ATP release, the role of P2X7R as a releasing channel for other gliotransmitters has been studied and a crucial advantage in these studies has been the use of radiolabeled tracers. GABA release has been studied in the RBA-2 astrocyte cell line where it has been shown that BzATP and ATP are able to induce the release of [^3^H]GABA in a concentration-dependent manner. This release was not blocked by the specific GABA transporter inhibitor nipecotic acid while it was blocked in the absence of Cl^−^ or HCO_3_^−^ [95]. This is because Cl⁻ or HCO₃⁻ ions are either necessary for the proper function of GABA-permeable anion channels or provide the electrochemical driving force required for GABA release. In primary mouse cortical astrocytes, it has been demonstrated that glutamate may also be released through P2X7R. It was shown that in basal conditions, 10–20% of the total radiolabeled glutamate was released, while upon high ATP, or BzATP, exposure glutamate release increased up to 40 and 90%, respectively, and the release was blocked by a short preincubation with the selective P2X7R antagonist oATP. Moreover, glutamate release was potentiated in divalent ion-free medium, a condition that favors P2X7R opening [96]. Recently, a similar approach has been used to show that astrocytic P2X7R plays a role also in D-serine release. D-serine is a co-agonist of the N-Methyl-D-Aspartate (NMDA) glutamate receptor and it may be released through a Ca^2+^-dependent mechanism by astrocytes, contributing to the setting of long-term potentiation [97]. Pan and co-workers loaded primary astrocytes with [^3^H]D-serine and demonstrated that in the presence of high concentration of eATP (1–2 mM), D-serine was released by astrocytes, a result also obtained by cell stimulation with high BzATP concentrations. The authors further showed that astrocytes release D-serine through the Panx1 hemichannel in a Ca^2+^-independent way, following the formation of the P2X7R-Panx1 complex [98]. Most of the works studying gliotransmitter release following P2X7R activation have been done in cell culture. Carmignoto’s group was the first investigating glutamate release by astrocytic P2X7R in rat hippocampal brain slices. The authors showed that BzATP (100 μm) triggers the astrocytic release of glutamate, which in turns elicits both transient, slow inward currents (SICs) [99] and tonic currents in CA1 pyramidal neurons. By using P2X7R inhibitors, oATP and BBG, it was shown that while SIC generation was not blocked, tonic currents were completely abolished in all neurons tested, therefore indicating that P2X7R activation results in glutamate release by astrocytes that mediates neuronal tonic currents in CA1 region of the hippocampus [100]. The role of activated astrocytic P2X7R has been studied also in mouse brain slice preparations of hippocampal CA1 and CA3 regions. P2X7R was selectively activated by using high BzATP concentration (1mM). This high concentration was needed because astrocytes from mouse pre-frontal cortex [101] are less sensitive to BzATP with respect to rat brain slice preparations. The authors demonstrated that in the CA1 region, P2X7R activation in astrocytes induces GABA release, which modulates pyramidal neuron activity. In contrast, in the CA3 region, BzATP primarily activates P2X7R on oligodendrocyte-like NG2 glial cells, rather than astrocytes; however, the gliotransmitter released by NG2 cells upon activation was not identified [102]. eATP is considered an important signal not only in neuron-astrocyte crosstalk but also for astrocyte-to-astrocyte Ca^2+^-mediated communication [103, 104], with astrocytes responding to eATP with a propagating wave of intracellular Ca^2+^ rise [103]. Ballerini and co-workers argued that the P2X7R antagonist oATP blocks ATP-induced Ca^2+^ increases in primary astrocytes, supporting a key role of P2X7R in this process [105]. It is worth mentioning that this might be true in the presence of high concentrations of eATP, needed to activate P2X7R, while in physiological conditions, when eATP concentrations are lower and P2X7R mostly inactive, other molecular mechanisms could be on action [106, 107].

### Astrocytic P2X7R and eATP Sensitivity

Astrocytes respond to eATP through different mechanisms that may be related to the engagement of both metabotropic and ionotropic purinergic receptors. Although in physiological conditions P2X7R needs an high eATP concentration to be activated, astrocytic P2X7R affinity to eATP might be increased in the presence of nicotinamide adenine dinucleotide (NAD^+^), via an ecto-ADP-ribosyltransferase 2 (ARCT-2)-catalyzed ADP-ribosylation of the P2X7R itself, a mechanism already demonstrated to be present in peripheral T cell [108, 109]. A similar pathway is present in astrocytes in brain ischemic tolerance condition, which represents a neuroprotective mechanism established after a noninvasive preconditioning ischemic episode (PC) leading to a resilient condition to lethal ischemia [110]. In mice, after a PC ischemic episode, P2X7R results upregulated in astrocytes [111] and eATP levels increase, although still in the nM range, i.e. not sufficient to activate P2X7Rs. After the PC episode, however, a substantial increase in ARCT-2 expression was found in astrocytes, but not in microglia, supporting the hypothesis of a possible ADP-ribosylation of P2X7Rs in these cells, leading to receptor sensitization to lower eATP concentration. On the other hand, it has been previously shown that high concentrations of ATP (mM range) lead to an increase of HIF-1α in astrocytes from WT mice, while this increase is absent in astrocytes from P2X7RKO mice [112]. On this line, in the presence of NAD^+^ the increase in HIF-1α can be obtained also with lower ATP concentrations, thus confirming the existence of an alternative pathway for P2X7R activation in astrocytes [113].

### Astrocytic P2X7R and Inflammation

As for microglia, high concentrations of eATP lead to the activation of the NLRP3 inflammasome through P2X7R stimulation in astrocytes. This pathway has been recently studied in the context of mechanical strain, i.e., transient increase of the intraocular pressure (IOP), both in vivo and in primary optic nerve astrocytes. Firstly, it has been shown in vivo that the intravitreal injection of the P2X7R antagonist BBG, before the IOP rise, is sufficient to prevent the increase in IL-1𝛽 release, and similar results were obtained also in P2X7RKO mice. Moreover, experiments in primary optic nerve astrocytes also showed that mechanical strain increases IL-1𝛽 levels, and this increase was prevented in the presence of the soluble ectoATPase apyrase, or by the inhibition of the Panx1 channel, pointing to a central role of P2X7R in priming IL-1𝛽 release also in astrocytes [114]. Noteworthy, a similar mechanism occurs also in the context of chronic strain [115]. Interestingly, upon a transient increase of IOP there is also a shift in astrocyte phenotype towards a reactive state, as revealed by an upregulation of *Gfap*,* Steap4*,* Vim*,* SerpinA3N*, and *Aspg* genes [116], all genes related to astrocyte reactivity [117]. A similar phenotype can be reached also by using BzATP, and it is partially absent in P2X7RKO mice, being only part of the aforementioned genes upregulated in these animals [116]. These results point to a crucial role of P2X7R in regulating astrocyte reactivity and cytokine release in the presence of high eATP concentrations. By using LPS as proinflammatory stimulus, further ATP stimulation of astrocytes may also mediate EV release, as it has been shown in primary cultured astrocytes from WT mice. P2X7R plays a key role also in this context, since astrocytes from P2X7RKO mice showed a reduced EV release, a phenotype that could be replicated in primary astrocytes from WT mice pre-incubated with GSK1482160, a CNS-penetrant P2X7R inhibitor [118]. It should be mentioned that this study has been performed by taking into consideration primary astrocytes that have been previously activated with LPS, while no experiments with only ATP stimulation were performed.

### Microglial and Astrocytic P2X7R in Alzheimer’s Disease

AD is a progressive neurodegenerative disorder characterized by amyloid-β (Aβ) plaques deposition, tau hyperphosphorylation and chronic neuroinflammation. The latter process is nowadays a hot topic in AD research, with microglia and astrocytes recognized as pivotal players [119, 120]. Noteworthy, several behavioral studies have demonstrated that P2X7R inhibition not only reduces pathological lesions in AD models but also enhances synaptic plasticity and alleviates associated cognitive impairments [121–124]. When evaluating the role of P2X7R and microglia in AD pathology, several factors must be considered, including the experimental AD model employed and the disease stage under investigation. Moreover, P2X7R is characterized by several genetic polymorphisms that can affect receptor function [125]. For instance, two of the most investigated *P2RX7* SNPs in AD patients are 1513 A > C, which is associated with a loss-of-function receptor and an anti-inflammatory profile, and 489 C > T, a gain-of-function variant that enhance Aβ clearance by promoting microglia phagocytosis [15, 68, 126]. Despite these aspects, it is well established that P2X7R is upregulated in microglia of various AD models, as well as in postmortem brains of AD patients [47, 127, 128]. Along with this, also in primary mouse microglia cultures, Aꞵ_1−42_ treatments elicit an increase in ATP release and IL-1ꞵ secretion, which is instead prevented in similarly treated microglial cells obtained from P2X7RKO mice [129]. In AD mouse models, P2X7R expression is markedly increased in microglia surrounding Aβ plaques, where P2X7R activation promotes the production of superoxide [47, 127, 128, 130], and its expression correlates with increasing Aꞵ plaque burden [128]. During neuroinflammation, the activation of P2X7R by eATP promotes microglia migration towards Aꞵ plaques, particularly during late AD stages, contributing to the accumulation of reactive microglia around these structures [130]. Interestingly, pharmacological blockade or genetic deletion of P2X7R enhances microglia phagocytosis and clearance of Aβ_1–42_ peptides and significantly reduces both the number and the size of Aβ plaques [121, 131, 132]. Additionally, P2X7R activation stimulates the secretion of tau-containing exosomes from microglia, thereby contributing to tau pathology [122]. On the same line, increased P2X7R levels worsen tau pathology in P301S mice [124] and NLRP3 inflammasome activation can also drive tau hyperphosphorylation and aggregation through the IL-1β signaling pathway [133]. Moreover, both the P301S and the APP/PS1 mouse models show an increase in P2X7R expression in hippocampal and cortical astrocytes [121, 134], suggesting a potential role of astrocytic P2X7R in AD progression. In primary cultures from WT astrocytes, fibrillar Aβ_1–42_ led to an increased release of the CCL3 chemokine. Chemokines contribute to inflammatory processes and to neurodegeneration, acting on immune cell recruitment and activation [135–137]. Interestingly, CCL3 release was blunted in P2X7RKO primary astrocytes. Although the central role of astrocyte in AD has been extensively studied [138] to the best of our knowledge, no other studies tried to clarify whether and how P2X7R impacts on astrocyte function in the AD context.

### Microglial and Astrocytic P2X7R in Epilepsy

Epilepsy is a neurological disorder characterized by recurrent spontaneous seizures due to abnormal brain electrical activity. It affects individuals of all ages with multifaceted severity and symptoms. eATP seems to be involved in epileptic seizures. Indeed, high quantities of ATP has been reported in human hippocampal slices from drug-resistant epilepsy patients, during ictal discharges [139]. Likewise, kainic acid (KA)-induced seizures in mice have been shown to enhance ATP release in comparison to vehicle-treated controls [140]. Many cellular and intracellular pathways induced by P2X7R activation are altered in epilepsy, and the targeting of NLRP3 inflammasome, and the consequent release of IL-1β, has been shown to be a promising anti-seizure treatment [141]. However, although P2X7R expression is generally increased in both epilepsy experimental models and patients [142, 143] its role in the generation, severity, and frequency of seizures remains controversial. Indeed, while some studies have reported anticonvulsant effects of the pharmacological blockade, or genetic deletion, of P2X7R [35, 144–146] others have found no significant effect [147, 148], or have even suggested a potential proconvulsant role for the receptor [149, 150]. Interestingly, microglia depletion leads to a decreased seizure threshold in mice [151]. This proconvulsant role of microglia seems to be related to P2X7R: a recent study showed that, in mice, an increased P2X7R function specifically on microglia reduces responsiveness to anti-convulsant drugs during status epilepticus (SE) [152]. Related to this, Alves and colleagues employed a conditional Cre-LoxP system in murine models of epilepsy, to delete the exon 2 of the *P2RX7* gene in either microglia or neurons. Deletion of the receptor in microglia led to a reduction in inflammation and in the expression of pro-convulsant cytokines, such as IL-1β, and a less severe epileptic phenotype. This phenotype was accompanied by an increase in anti-inflammatory cytokines, such as IL-10, suggesting a shift of microglia towards a neuroprotective status. On the other hand, neuronal deletion of P2X7R resulted in increased seizure susceptibility, reduced GABA levels, and higher neurodegeneration, indicating a protective role for the receptor in neurons during epilepsy [153]. The role of astrocytic P2X7Rs has been examined in a mouse model of KA-induced SE. Selective deletion of P2X7R in hippocampal astrocytes, using shRNA vectors, led to improved cognitive performance compared to KA-treated control mice. In contrast, deletion of P2X7R in neurons did not alleviate cognitive impairments, underscoring a specific contribution of astrocytic P2X7R to KA-induced cognitive deficits. KA-induced SE was associated with increased Ca²⁺ activity in both hippocampal neurons and astrocytes. The authors propose that this elevated Ca²⁺ signaling triggers the release of ATP and glutamate. While glutamate is critical for long-term potentiation (LTP) and memory formation, ATP may activate astrocytic P2X7Rs, potentially impairing cognitive processes either directly or through its metabolite, adenosine. Consistent with this, pre-treatment with A2AR or P2X7R antagonists prior to KA administration prevented the KA-induced suppression of LTP in mice expressing P2X7R [154].

## Discussion

Since their discovery in 1976, many studies provided compelling evidence for the role of purinergic receptors in CNS physiopathological conditions. Among them, P2X7R has been mainly studied in pathological conditions, when it is activated by high eATP concentrations. In the previous chapters, we focused on the role of microglial and astrocytic P2X7R. Microglial P2X7R have been extensively studied, mainly in primary cultures, often drawing on experimental approaches and mechanistic insights from peripheral macrophage research. From this foundation, the M1/M2 polarization framework for microglia was proposed. However, we support the view that microglia exist in a continuum dynamic spectrum of activation states that cannot be adequately captured by this binary classification [155]. In addition to their classical role as immune sentinels of the brain, recent studies have highlighted microglial contributions to the modulation of neuronal activity - promoting neuronal synchrony [156] and suppressing hyperexcitability [151]. Given that altered neuronal excitability is a hallmark of numerous neurological disorders, including AD and epilepsy, elucidating the role of microglial P2X7R in these contexts is of significant interest. In contrast to microglia, the role of astrocytes in synaptic transmission is well established. However, emerging evidence indicates that this role is far more complex than previously appreciated, unveiling new layers of regulation and dynamic interactions within the tripartite synapse [84]. In this context, astrocyte heterogeneity has become a major focus of current research. This diversity is particularly relevant to the expression and function of P2X7R. Indeed, astrocytic functional and morphological heterogeneity may become even more pronounced under neuroinflammatory conditions - precisely when P2X7R is likely activated. Yet, the impact of P2X7R activation across different astrocyte subtypes and pathological contexts remains largely unexplored. While the use of broad-spectrum pharmacological P2X7R antagonists and global P2X7RKO mice has been instrumental in uncovering key roles of the receptor, future studies would greatly benefit from strategies that allow temporally controlled gene deletion or overexpression, particularly within defined brain cell subpopulations.

## Data Availability

No datasets were generated or analysed during the current study.
